# Changes functional prediction of ear canal flora in chronic bacterial otitis externa

**DOI:** 10.3389/fcimb.2024.1434754

**Published:** 2024-10-23

**Authors:** Tingting Duan, Zhiqun Li, Xiaoyong Han, Qichao Hong, Yunan Yang, Jinren Yan, Chengliang Xing

**Affiliations:** ^1^ Department of Otolaryngology, Head and Neck Surgery, The First Affiliated Hospital, Hainan Medical University, Haikou, China; ^2^ Department of Otolaryngology, Linping District Hospital of Traditional Chinese Medicine, Hangzhou, China; ^3^ Department of Otorhinolaryngology-Head and Neck Surgery, Hainan General Hospital (Hainan Affiliated Hospital of Hainan Medical University), Haikou, China

**Keywords:** chronic otitis externa, microbiome, bacteria, OTU, function prediction

## Abstract

**Objective:**

To investigate ear canal microflora’s structure, composition and function in patients with chronic bacterial otitis externa.

**Methods:**

A case-control study design method was used to collect the ear canal secretions from 14 patients with chronic bacterial external otitis (CB group) and 14 healthy controls (H group) treated in the Department of Otolaryngology and Head and Neck Surgery in the First Affiliated Hospital of Hainan Medical University. 16S rRNA high-throughput sequencing technology was used to sequence the ear canal microflora’s V3 ~ V4 region gene amplification products in the participating population. The α diversity of ear canal microflora in 2 groups was analyzed. Based on the weighted Unifrac distance, principal coordinate analysis was performed to compare the β diversity of ear tract microflora between the two groups. The differences in ear microflora at phylum and genus levels were analyzed. PICRUSt2 function prediction and BugBase phenotype prediction were also performed.

**Results:**

α diversity analysis showed that the diversity and richness of auricular microflora in the CB group were significantly lower than those in the H group. β diversity analysis showed that there were some differences between the two groups. At the phylum level, the relative abundance of *Bdellovibrionota, Campylobacterota, and WPS-2* in the microbiota of patients in the CB group was significantly lower than that in the H group, and the differences were statistically significant. At the genus level, the relative abundance of *Pseudomonas*, *Acinetobacter*, *Pelomonas*, *Sphingomonas*, *Bradyrhizobium*, *Brevundimonas*, *Enhydrobacter*, *Actinomyces*, *Paracoccus* and *Chryseobacterium* in the ear canal of Group H is significantly higher than that of Group CB. Functional prediction of PICRUSt2 suggests that amino acid biosynthesis and bacterial microbiota may be related. In BugBase phenotypic prediction, the contribution of aerobic phenotype in group CB was significantly lower than that in group H.

**Conclusion:**

The diversity and abundance of the ear canal flora of patients with chronic bacterial otitis externa were significantly lower than those of the healthy population, and their bacterial colony structure was significantly altered. Dysbiosis of the ear canal flora may be an important cause of chronic bacterial otitis externa.

## 1 Introduction

All organs of the human body have a large number and variety of microorganisms (Postler and Ghosh, 2017). There are different types and numbers of microorganisms in the human external auditory canal, and the normal flora can form a natural barrier against the invasion of foreign pathogenic bacteria. In recent years, ear canal diseases have shown a rising trend, and the extensive use of antibiotic drugs and immunosuppressants has constituted a more obvious impact on microorganisms, which has damaged the antagonistic function of the normal flora (Lasisi et al., 2007; Costello et al., 2009; Ren et al., 2018). About 10% of people may experience external otitis at some point in their lives, making it one of the most prevalent illnesses in the head and neck area (Simon, 2020). Chronic bacterial otitis externa is a common otological disease closely related to changes in the micro-ecosystem of the external auditory canal, which is more difficult to cure and prone to recurrence than simple acute bacterial otitis externa, and it is a challenging problem in otological diagnosis and research. The study of environmental bacteria in the ear canal is the basis for understanding the pathogenesis of bacterial otitis externa.

The enormous impact of the human microbiome on health and disease has led to it being referred to as the “second genome” (Grice and Segre, 2012). Disease in the host can result from dysbiosis, a disruption in the microbiome brought on, for example, by antimicrobials, damage to the epithelium, or altered immune defense systems (Walker and Lawley, 2013). It is postulated that decreasing environmental biodiversity might have an impact on human microbiota, leading to a rise in the occurrence of inflammatory disorders such as allergies and asthma (von Hertzen et al., 2011; Haahtela, 2019). Changes in the gut microbiota have been linked to conditions including colorectal cancer, ulcerative colitis, Parkinson’s disease, and primary sclerosing cholangitis (Marchesi et al., 2011; Bajer et al., 2017; Aho et al., 2019; Scott et al., 2019). Localized skin ecological dysregulation and loss of microbial diversity have been associated with several skin diseases, such as chronic otitis externa, acne vulgaris, and psoriasis (Codoñer et al., 2018; Nawaz et al., 2023).

Microscopic examination and isolation culture methods have limited ability to detect flora, and can only detect less than 1% or even less of the actual number of flora in the microenvironment of the external ear canal, so they cannot truly reflect the status of the flora of the external ear canal, nor can they analyze the relationship between different strains. With the continuous development of molecular biology, a large number of scholars have applied gene technology to conduct extensive research on the four major human flora (namely oral, stomach, intestinal, and vaginal flora) from different perspectives (Lamont et al., 2011; Lynch and Pedersen, 2016), making people have a deeper understanding of human flora. However, until now, the specific changes of the microflora in the external ear canal environment and the relationship between them and the onset of external otitis is not clear, and an in-depth understanding of the structure of the external ear canal microflora is an important theoretical basis for preventing the occurrence of chronic external otitis(bacteria).

Therefore, this study collected samples of ear canal secretion (cerumen) from both healthy individuals and those with chronic bacterial otitis externa. Total DNA was extracted from the cerumen and the V3+V4 region of bacterial 16S rRNA was sequenced for analysis. The study aimed to investigate the differences in bacterial diversity between normal individuals and patients, as well as functional changes in bacterial flora. These findings may provide a basis for preventing chronic bacterial otitis externa and improving clinical diagnosis and treatment.

## 2 Materials and methods

### 2.1 Study subjects and sample collection

The study was conducted by the principles of the Declaration of Helsinki. The study protocol was approved by the Ethics Committee of the First Affiliated Hospital of Hainan Medical University (approval number: 2024-KYL-123). All samples (cerumen) were taken from the outpatient Department of Otolaryngology, Head and Neck Surgery, the First Affiliated Hospital of Hainan Medical University. The study was divided into two groups: chronic bacterial otitis externa (CB) and healthy (H). In different groups, only unilateral ear cerumen was collected for subsequent analyses and studies.

The inclusion criteria for the CB group are as follows: 1) adults over the age of 18; 2) The disease has been tested for more than three months; 3) The clinical and pathological examination showed bacterial otitis externa; 4) No treatment. Exclusion criteria: 1) suffering from ear disease; 2) Autoimmune diseases; 3) Oral or topical antibiotics. All external auditory canal secretions or cerumen are collected into a sterile container using sterile cotton swabs and sterile tweezers (collect as many samples as possible without affecting the patient).

Inclusion criteria for the H group) adults over the age of 18; 2) No symptoms or history of otitis externa, otitis media, or other ear diseases; 3) No history of ear surgery; 4) No history of long-term use of antibiotics or steroid medications; 5) Willing and able to comply with all requirements and regulations of the study. Exclusion criteria for the H group) Symptoms or history of otitis externa, otitis media, or other ear diseases; 2) History of ear surgery; 3) History of long-term use of antibiotics or steroid medications. 4) History of allergic diseases or immune system disorders.; 5) Unable to comply with all requirements and regulations of the study.

### 2.2 DNA extraction and PCR amplification

Genome DNA extraction kit (Omega Bio-tek, Georgia, U.S.) was used to extract genomic DNA from microbial samples in ear canal contents, and the template was used for PCR amplification after passing the test. Using the extracted genomic DNA as template, 16S rRNA gene V3 to V4 universal primers 338F (5’ACTCCTACGGGAGGCA-GCA-3’) and 806R (5’- GGACTACHVGGGTWTCTAAT-3’) were used as upstream and downstream primers for PCR amplification (Liu et al., 2016). The PCR reaction mixture contained 10 ng of template DNA, ddH2O to a final volume of 20 µL, 4μL 5 × Fast Pfu buffer, 2μL 2.5 mM dNTPs, 0.8μL each primer (5 μM), and 0.4μL Fast Pfu polymerase. The cycling conditions for the PCR amplification were as follows: a three-minute initial denaturation at 95°C, 27 cycles of denaturing at 95°C for 30 s, annealing at 55°C for 30 s, and extending at 72°C for 45 s, followed by a single extension at 72°C for 10 min, and ending at 4°C. Three duplicates of each sample were amplified. After being removed from a 2% agarose gel, the PCR product was purified. Afterwards, QuantusTM Fluorometer (Promega, USA) was used to quantify. Purified amplicons were pooled in equimolar amounts and paired-end sequenced on an Illumina PE300 platform (Illumina, San Diego, USA) according to the standard protocols by Majorbio Bio-Pharm Technology Co. Ltd. (Shanghai, China). The sequencing process was completed by Shanghai Meiji Biomedical Technology Co., LTD.

### 2.3 Data processing

Raw FASTQ files were de-multiplexed, quality-filtered with fastp, and merged using FLASH. Reads with an average quality score <20 over a 50 bp sliding window or ambiguous characters were discarded, and sequences <50 bp were removed. Overlapping sequences longer than 10 bp with a maximum mismatch ratio of 0.2 were assembled; unassembled reads were discarded. Sequences were clustered into operational taxonomic units (OTUs) using UPARSE 11 with 97% similarity (Edgar, 2013), and chloroplast sequences were manually removed. Sequences were rarefied to 20,000 per sample to minimize sequencing depth effects, ensuring 99.09% coverage.

### 2.4 Statistical analysis of data

Using Mothur v1.30.2 (Schloss et al., 2009), rarefaction curves and alpha diversity indices, such as Chao1 richness, Shannon index, Good’s coverage, Ace index, and Sob index, were computed based on the OTU data. By utilizing the Vegan v2.4.3 software and principal coordinate analysis (PCoA) based on Bray-Curtis dissimilarity, the similarity between the microbial communities in various samples was ascertained. The linear discriminant analysis (LDA) effect size (LEfSe) (http://huttenhower.sph.harvard.edu/LEfSe) was performed to identify the significantly abundant taxa (phylum to genera) of bacteria among the different groups (LDA score > 2.5, *P* < 0.05) (Segata et al., 2011). After drawing a flat according to the minimum number of sample sequences, a Wayne diagram was used to compare the differences in species distribution between the two groups; the dominant species composition of the two groups at the phylum and genus levels was analyzed using the community Bar diagram; and the relative abundance of the two groups of bacterial flora was analyzed for the significance of differences, to obtain information on the species with significant differences between the two groups; and finally, the ear canal of the two groups was analyzed for functional and phenotypic predictions of the OTUs of the two groups, respectively, based on the PICRUSt2 (Douglas et al., 2020) and the BugBas (Kang et al., 2022) databases. Functional and phenotypic prediction of the OTUs of the microflora of the two groups based on the PICRUSt2 and BugBase databases, respectively, and the relative abundance of genera of the relevant phenotypes were selected for further analysis. The experimental data were compiled using Microsoft Excel 2023 software; the Wilcoxon rank-sum test was used for significance analysis; *P*<0.05 indicates a significant difference, *P*<0.01 indicates a highly significant difference and *P*>0.05 indicates a non-significant difference.

## 3 Result

### 3.1 Sequencing depth and operation taxon OTU

**Figure 1:**
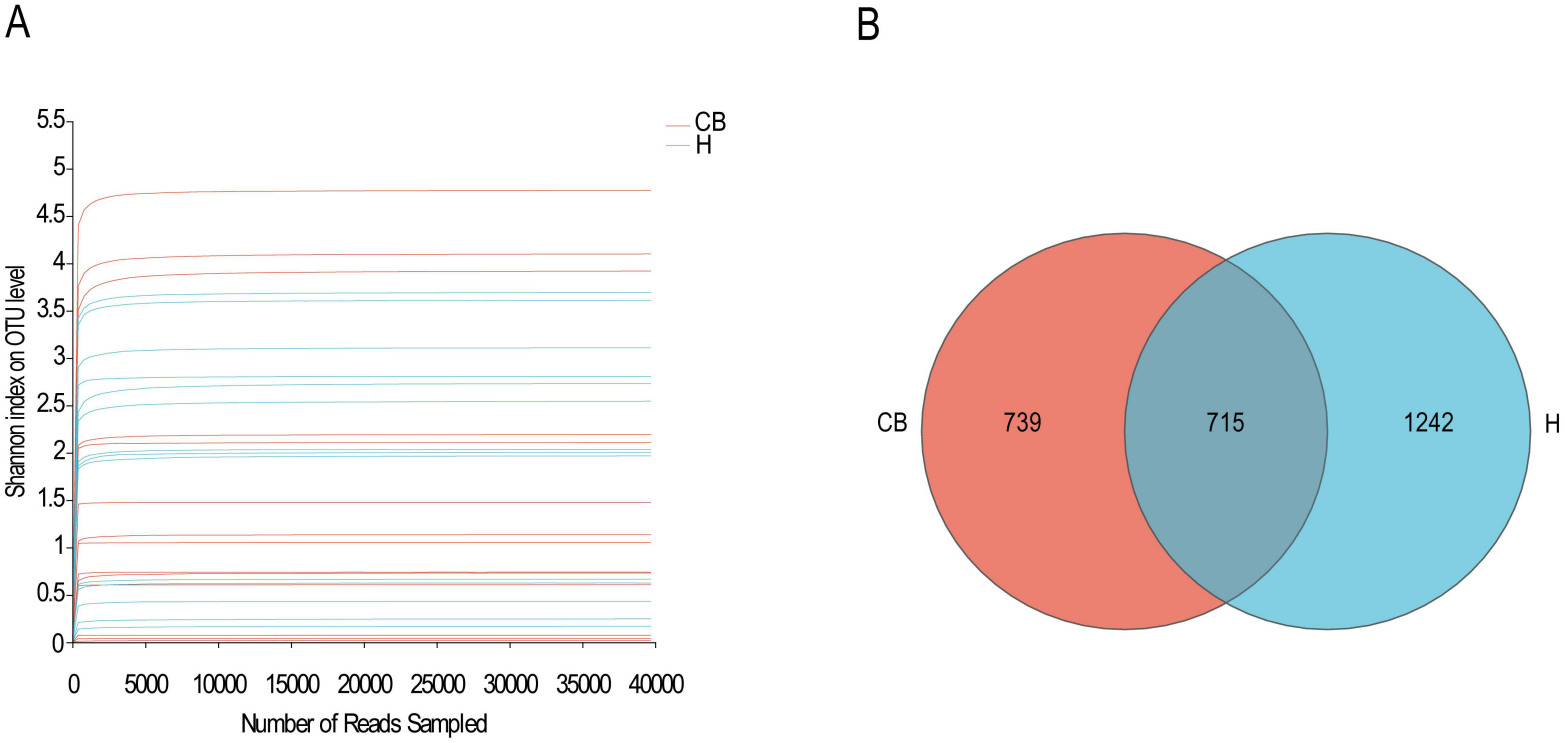
Species sequencing and composition. **(A)** The Shannon-Winner curve. **(B)** Venn diagram in OTU level between H and CB people investigated.

In this study, 14 specimens were obtained from both CB and H groups for subsequent analyses, and high-throughput sequencing was performed for the V3+V4 region of the bacterial DNA, and the sequences obtained from each sample ranged from 41596 to 280966, with the number of bases ranging from 24120756 to 79825645 bp, and the average sequence length was 400 bp, which met the sequencing requirements of the Illumina Miseq PE300 platform (the average sequence length should be greater than 200 bp), as shown in **Supplementary Table S1**. As shown in **Figure 1A**, the curves of all samples gradually flattened as the sequencing depth increased. The curve tends to be flatter, indicating that it is difficult to detect new OTUs with increasing sequencing depth, and the sequencing results have adequately reflected the diversity of current samples. The Veen plot of OTUs in the healthy and diseased groups is shown in **Figure 1B**. Bacterial overlap amounted to 715 OTUs, which was close to 30% of the total OTUs in the samples of the two groups, and reached 50% of the diseased group, indicating that the majority of the bacteria that the diseased group possessed were found in the ear canal cerumen of the healthy group, while the healthy group had a greater number of OTUs of more types than the diseased group.

### 3.2 Analysis of bacterial diversity in the ear canal

**Figure 2:**
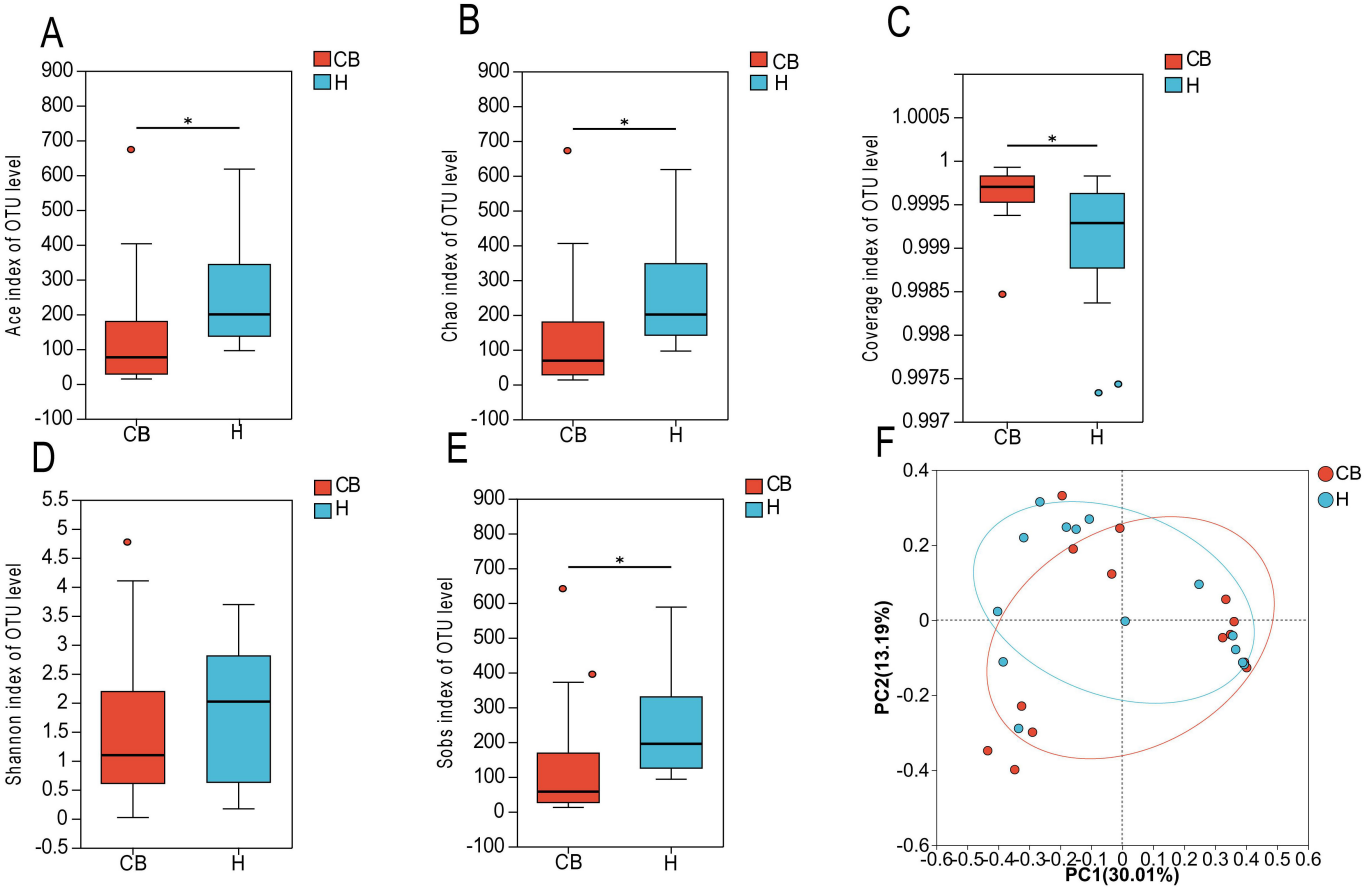
Species diversity analysis. **(A)** ACE index of OTU level. **(B)** Chao index of OTU level. **(C)** Coverage index of OUT level. **(D)** Shannon index of OTU level. **(E)** Sobs index of OTU level. **(F)** βdiversity. *(*p* < 0.05).

In this study, the 16S rRNA sequencing of the bacterial groups was analyzed for diversity. **Figure 2C** shows that the coverage index of both groups exceeded 0.999, indicating that the sequencing depth was reasonable. coverage is commonly used in microbial 16S sequencing as an indicator of sequencing depth, and the closer the value is to 1, the more reasonable the sequencing depth is, and the sequencing depth has covered all the species in the samples. The results of a diversity nalysis Ace, Chao1, Sob, and Shannon indices at the OTU level showed a decreasing trend in the CB group compared to the H group, suggesting that the abundance and diversity of bacteria in the CB group was lower than that in the H group (**Figures 2A, B, D, E**). To further analyze the differences between the healthy and diseased groups, the samples of the two groups were subjected to statistical analysis of β diversity. The results of bacterial principal component analysis (PCA) showed that the sample points of group H and group CB partially overlapped, and there were some differences between the samples. This indicates that there are some changes in the community structure of bacteria in the ear canal environment of patients with chronic bacterial otitis externa (**Figure 2F**).

### 3.3 Structural composition of the bacterial community

**Figure 3:**
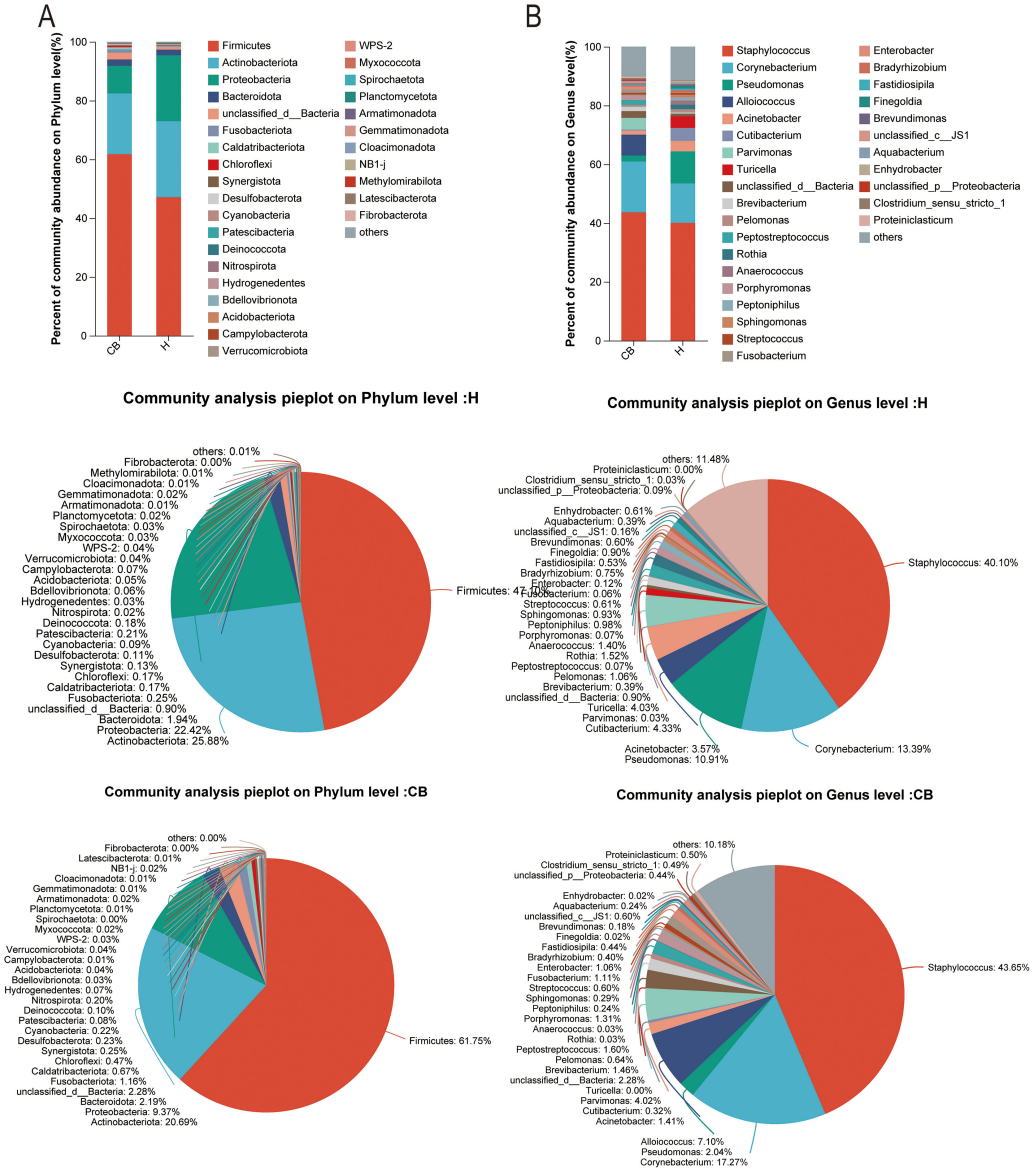
Analysis of community composition. Relative abundance of bacterial communities at phylum **(A)** and genus **(B)** level in the external auditory canal of healthy and diseased people investigated.

The results of community composition showed significant differences between the healthy and diseased groups at the phylum level of the bacterial community. Among the top ten abundance groups, *Firmicutes, Bacteroidota, Unclassified_d_bacteria, Fusobacteriota, Caldatribacteriota, Chloroflexi, Synergistota*, and *Desulfobacteriota* were more abundant in the CB group. However, *Actinobacteriota* and *Proteobacteriota* were more abundant in group H (**Figure 3A**). At the genus level, *Staphylococcus, Corynebacterium, Alloiococcus, Parvimonas, Unclassified_d_bacteria*, and *Brevibacterium* were more abundant in the CB group; *Pseudomonas, Acinetobacter, Cutibacterium*, and *Turicella* were less abundant in the H group (**Figure 3B**).

### 3.4 Analysis of species differences

**Figure 4:**
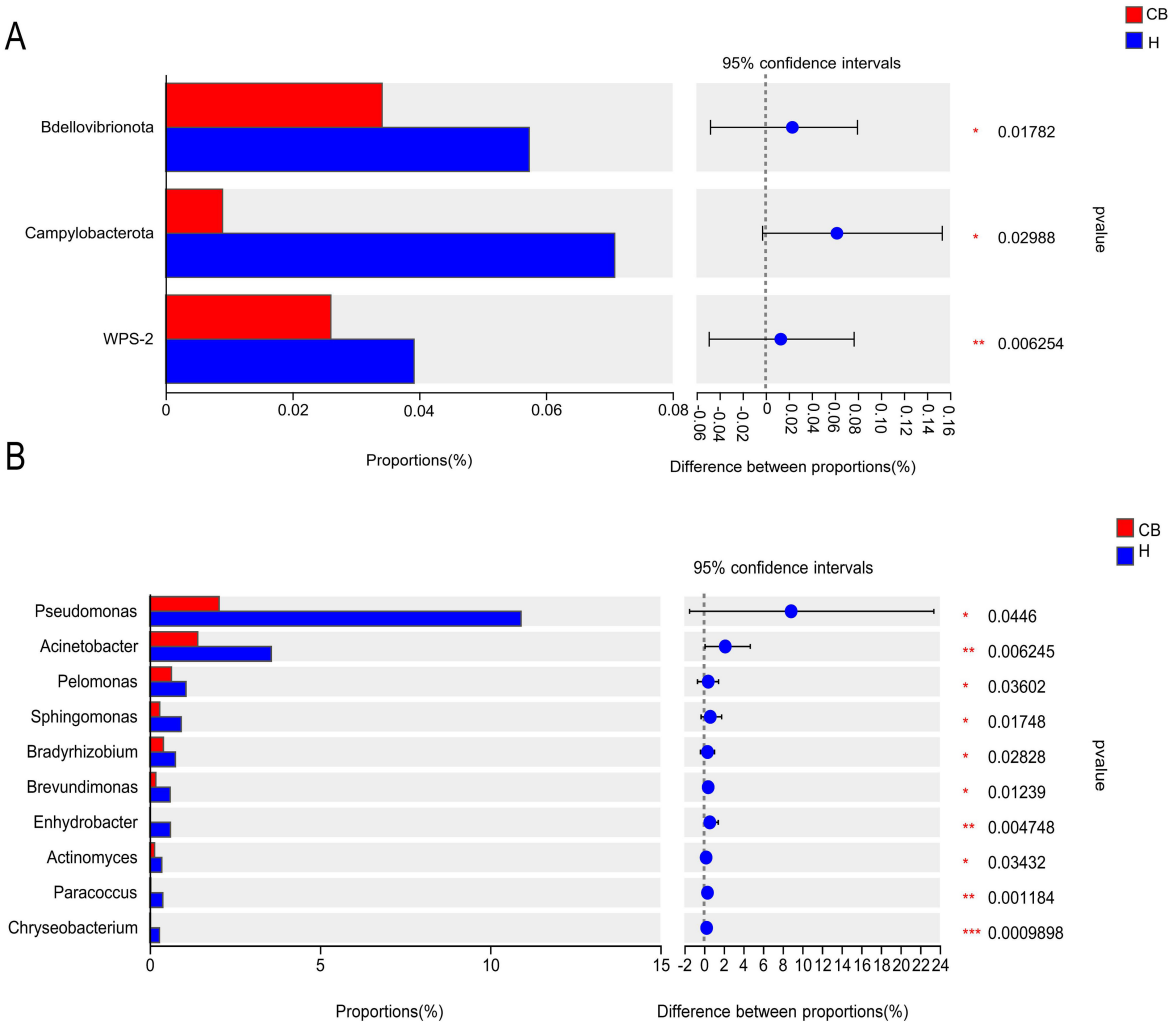
Results of the analysis of the ear canal flora in the two groups of patients. **(A)** Phylum level. **(B)** Genus level. *(*p* < 0.05); **(*p* < 0.01); ***(*p* < 0.001).

Wilcoxon rank-sum test was used to analyze the difference in intestinal flora between the two groups. The results showed that the relative abundance of *Bdellovibrionota, Campylobacterota*, and *WPS-2* in the ear canals of the CB group was significantly lower than that of the H group at the level of mycobacterial phylum (*P*<0.05) (**Figure 4A**). At the genus level (top 10 in relative abundance), as shown in **Figure 4B**, the relative abundance of *Pseudomonas, Acinetobacter, Pelomonas, Sphingomonas, Bradyrhizobium, Brevundimonas, Enhydrobacter, Actinomyces, Paracoccus* and *Chryseobacterium* in the ear canal of Group His significantly higher than that of Group CB (*P*<0.05).

### 3.5 Functional predictions suggest that amino acid biosynthesis and bacterial microbiota may be related

**Figure 5:**
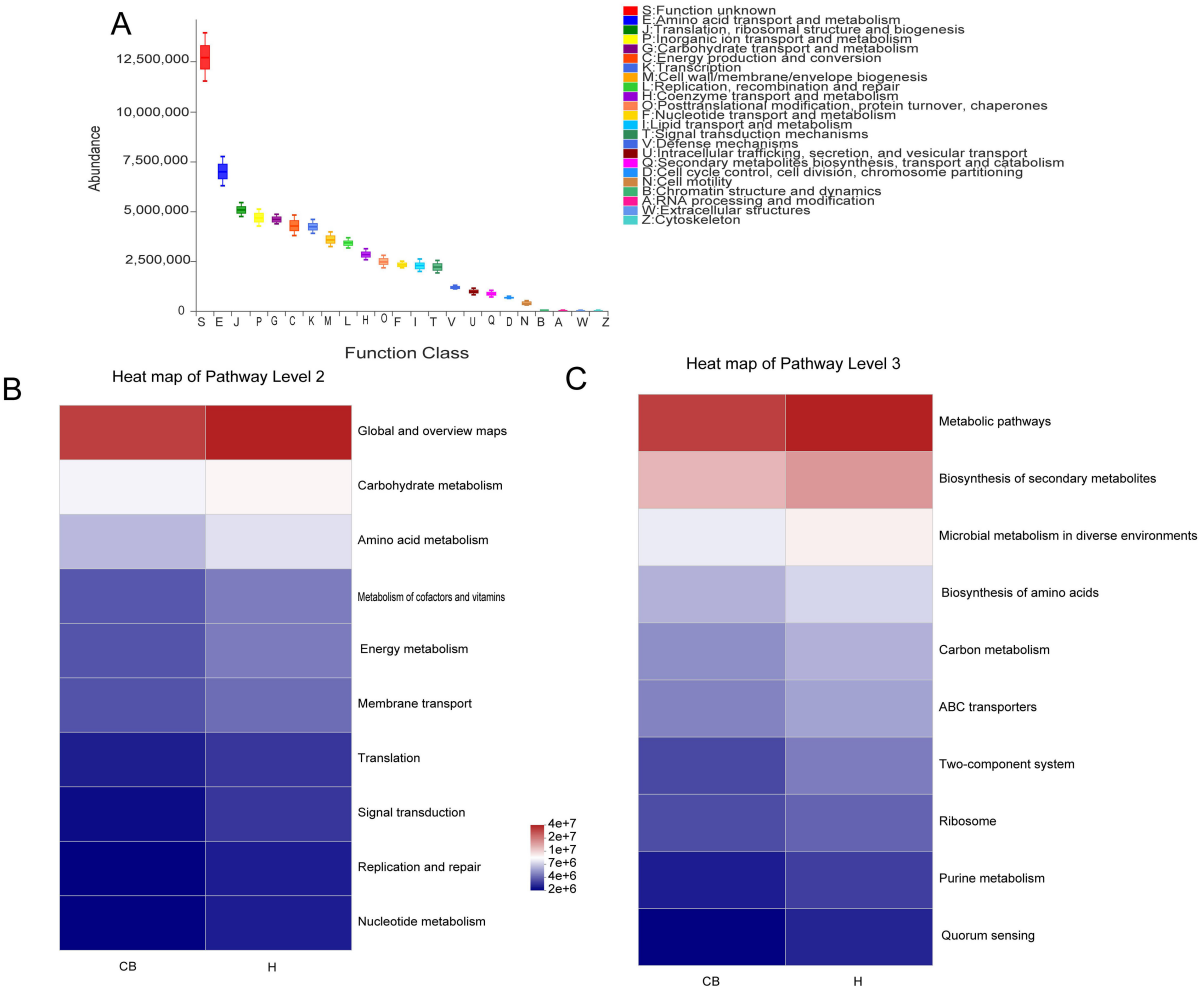
Analysis of the bacterial microbiome using functional prediction. **(A)** PICRUSt2 and the eggNOG database worked together to predict the role of the bacterial microbiota in various tissues. To predict the role of the bacterial microbiota in samples, PICRUSt2 merged with the KEGG database, displaying the results of the KEGG pathway at Levels 2 **(B)** and 3 **(C)**.

We projected the biological functions of the bacteria using the eggNOG and KEGG databases in conjunction with the 16S amplicon sequencing data(**Figure 5**). Combined with the eggNOG database, the function of bacteria was predicted to be mainly related to metabolism, among which amino acid metabolic abundance was the highest (**Figure 5A**). Similarly, the route of amino acid and nucleotide metabolism of carbohydrates is the main source of information on the activity of bacterial colonies, as suggested by the combination of 16S rRNA sequencing data and KEGG functional predictions (**Figure 5B**). The link between bacteria and the biosynthesis of amino acids, purine metabolism, and carbon metabolism could be further confirmed when combined with data from KEGG data at a deeper level (3rd Level) (**Figure 5C**).

### 3.6 BugBase phenotypic prediction of differential OTUs

**Figure 6:**
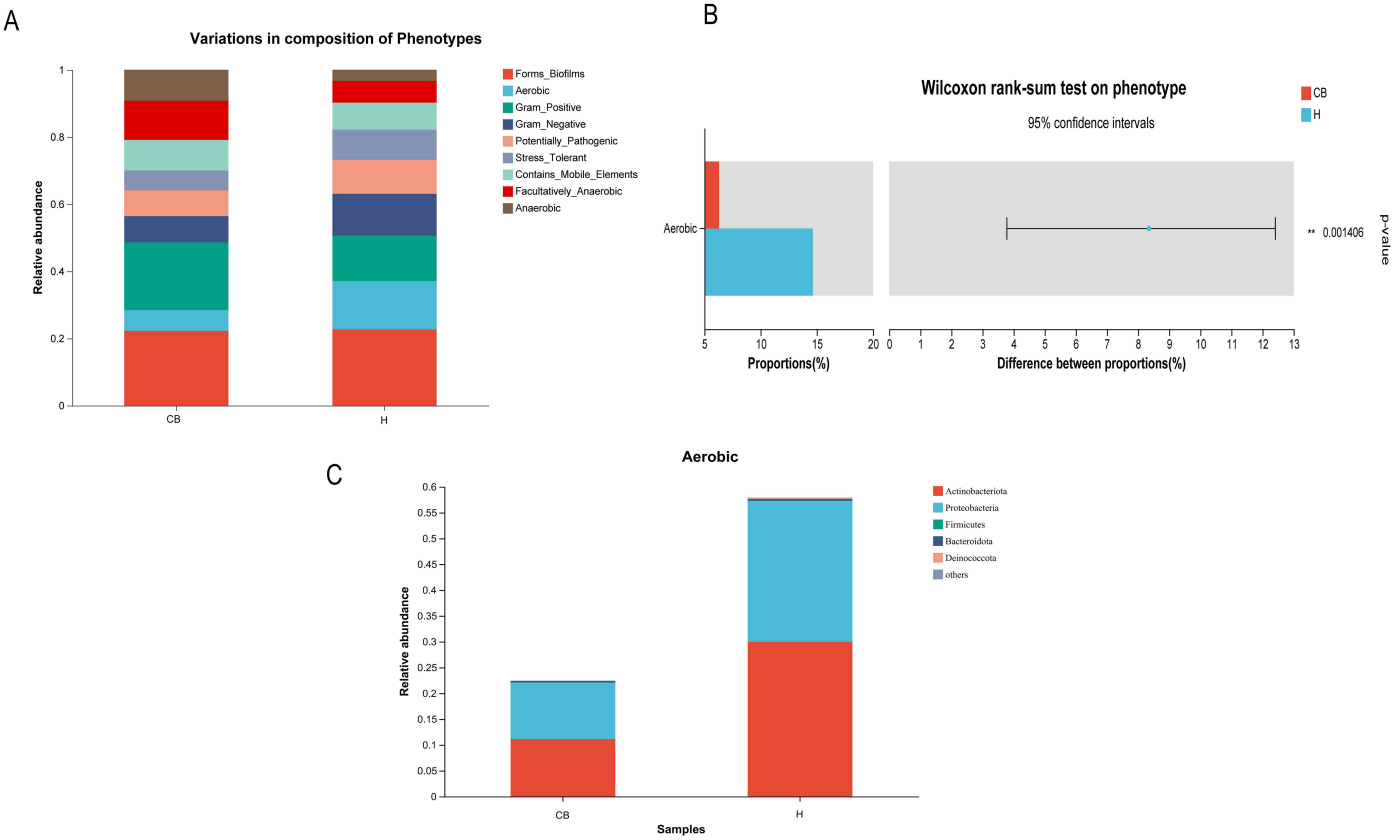
Results of Wilcoxon rank sum test for BugBase phenotypic prediction. **(A)** Distribution of nine potential microbial phenotypes in different groups. **(B)** Differential microbial phenotypes between the two groups. **(C)** Differential microbial phenotypes in the distribution of bacterial populations.

The results showed that a total of nine potential microbial phenotypes were detected, including forms_biofilms, aerobic, gram_positive, gram_negative, potentially, pathogenic, stress_tolerant,

contains_mobile_elements, facultatively_anaerobic, and anaerobic (**Figure 6A**). The contribution of the aerobic phenotype was significantly lower in the CB group compared to the H group (*p*<0.05). Other phenotypes saw no difference between the two groups (**Figure 6B**). Further analysis of the relative abundance of bacterial groups in this phenotype of aerobic showed that the *Actinobacteriota* and *Proteobacteria* were lower in the CB group than in the H group (**Figure 6C**).

## 4 Discussion

Chronic bacterial otitis externa is a form of chronic otitis externa, which, because of its complexity and ease of recurrence, puts a burden on the patient not only physically and mentally but also economically. It has been shown that the development of chronic bacterial otitis externa is closely related to dysbiosis of the microbiota (Wiegand et al., 2019). However, the relationship between microbiota and chronic bacterial otitis externa is less reported. In this study, we used 16S rRNA sequencing technology to analyze the V3-V4 regions of the ear canal flora of patients with chronic bacterial otitis externa and healthy controls, to compare the diversity of the ear canal microbiota, the differences in the composition of the flora and their functions in the two groups, and to explore the changing pattern of the bacterial flora in patients with chronic bacterial otitis externa.

In this study, significant differences in the diversity of ear canal flora were found between patients with chronic bacterial otitis externa and healthy controls. a diversity finding showed that the diversity and abundance of flora in patients with chronic bacterial otitis externa were significantly lower than that in the control group. In addition, the results of β diversity analysis also showed that the structural composition of the ear canal flora in patients with chronic bacterial otitis externa differed somewhat from that of healthy controls. This suggests that the microecology of the ear canal in patients with chronic bacterial otitis externa is disturbed, the diversity of the bacterial flora is reduced, the stability of the ear canal is disrupted, and the alteration of the microbiota of the ear canal may play a potential pathogenic role in chronic bacterial otitis externa.

In the study of external ear canal microbiota in patients with chronic external otitis and healthy individuals, the results of Lee JS (Lee et al., 2022) and colleagues have similarities with our study. At the phylum level, the abundance of microbial expression in each group from highest to lowest is consistently *Airmicutes, Actinobacteria, Proteobacteria, and Bacteroidetes*; while at the genus level, *Staphylococcus* predominates. Moreover, the trend in bacterial expression between the two groups tends to be consistent. However, there is a decrease in bacterial diversity, richness, and evenness in the patient group. Nevertheless, there are differences between the two studies. Lee JS and colleagues confirmed that fungi play a minimal role in disease regulation. In contrast, our study made more use of the eggNOG, KEGG, and BugBase phenotypic prediction databases to understand the changes in bacterial functions between different groups, providing a theoretical basis for future treatment options. These differences allow us to gain a more comprehensive understanding of the distinctions in external ear canal microbiota between patients with chronic external otitis and healthy individuals, offering more precise guidance for clinical treatment.

In this study, we found that the ear canal flora of patients with chronic bacterial otitis externa differed significantly from that of the healthy population in terms of both phylum and genus-level composition. *Staphylococcus* and *Corynebacterium* were abnormally elevated in the diseased group. *Staphylococcus* infections always depend on the bacteria breaking through the protective epithelial layer. For example, skin infections of the external auditory canal can develop from minor scratches in the skin and may become invasive (Stryjewski and Chambers, 2008). Among other things, *S. aureus* can also actively promote epithelial destruction, with the alpha-toxin cleaving the E-cadherin molecule primarily through activation of metalloproteinase structural domain protein 10 (ADAM10) (Inoshima et al., 2011; Inoshima et al., 2012). This mechanism disrupts adhesion junctions and destroys the actin cytoskeleton (Popov et al., 2015). In epithelial rupture, the success of *Staphylococcus* infection depends on the effective evasion of host defenses. *Staphylococci* invade organs and tissues by leaving their high concentration of cellular and humoral immune defense mechanisms in the bloodstream, where they form encapsulated abscesses (Cheung et al., 2021). *Corynebacterium* belongs to conditional bacterium, which can not only cause inflammation but also up-regulate the expression of TLR2, to potentially amplify and maintain the inflammatory response of human keratinocytes to TLR2 stimulation (Altonsy et al., 2020). It also upregulates the pro-inflammatory proteins il-1β, il-6, and CSF3 involved in the NF-κB pathway (Okuda et al., 2005). This can lead to the occurrence of external ear canal inflammation. In addition, we also found abnormal reductions in the number of some opportunistic pathogens, such as *Pseudomonas* and *Acinetobacter*. *Pseudomonas* is a common bacterium that can cause infection of the outer ear canal. When the outer ear canal is damaged or immune system function is reduced, *Pseudomonas* has the opportunity to enter the outer ear canal and multiply, leading to infection (Treviño González et al., 2021). At the same time, a variety of toxins can be produced, including exotoxins and endotoxins (Fitzgerald and Pastan, 1993; Horii et al., 2005). These toxins can damage the tissue of the outer ear canal, causing inflammation and pain. In addition, *Pseudomonas* has strong drug resistance and can resist the action of multiple antibiotics (Dimopoulos et al., 2020). This makes it more difficult to treat an external ear canal infection. *Acinetobacter*, as an opportunistic pathogen, possesses several characteristics that contribute to its ability to cause infections in the external auditory canal of humans. Its cell wall structure aids in resisting attacks from the host’s immune system (Wong et al., 2017). It forms biofilms to protect itself and promote metabolic activities (Mea et al., 2021). *Acinetobacter* secretes virulence factors that damage host cells and trigger inflammatory responses. Additionally, it exhibits resistance to a wide range of antibiotics (Lee et al., 2017). These factors collectively enable *Acinetobacter* to colonize and subsequently infect the human external ear canal. This may be due to the small sample size, different sampling seasons, and so on. It can be seen from the above that there are two sides of the flora. Within a certain range, the flora plays a role in inhibiting pathogenic bacteria, and beyond a certain threshold, it becomes pathogenic bacteria.

PICRUSt2 is a tool that predicts gene functions based on 16S rRNA data from microbial communities. This method helps researchers infer unobserved gene functions, providing deeper insights into the metabolic potential of microbial communities and their possible pathogenic mechanisms in the external ear canal. It is particularly valuable for analyzing changes in microbial functions and their roles in external ear canal diseases (Yang et al., 2023). In this study, we found that amino acid metabolism was altered significantly abnormal in the external auditory canal, where amino acids are the basis of protein synthesis and are essential for bacterial growth and reproduction (Ferro et al., 2018). If the amino acid metabolism of bacteria is reduced, this may lead to a limitation of growth and reproduction, which in turn affects the interaction between the bacteria and the host. Abnormal amino acid metabolism may affect the toxins or metabolites released by the bacteria. These toxins and metabolites can activate host immune cells, such as macrophages and neutrophils, triggering an inflammatory response (Jiang and Shi, 2021; Jiang et al., 2021; Song et al., 2022). A prolonged inflammatory response may lead to a chronic inflammatory state of the external auditory canal. This further supports the influence of changes in the ear canal microbiota on disease.

When performing BugBase analyses of the flora of chronic otitis externa versus healthy individuals, the abnormal reduction of *Actinobacteria* and *Aspergillus* was a noteworthy phenomenon. These two bacterial groups play an important role in the human microbial community, and their reduction may have a significant impact on the microbial balance in the ear canal. Firstly, *actinobacteria* are an important class of antibiotic-producing bacteria that produce a wide range of antibiotics that help inhibit the growth of pathogenic bacteria and maintain the stability of the microbial community. A decrease in *Actinomycetes* in the chronic bacterial otitis externa group may lead to diminished pathogen inhibition, making the ear canal more susceptible to pathogenic bacteria, which may exacerbate or prolong the inflammatory process (Fatahi-Bafghi, 2019). Second, *aspergillus* is also a common group of bacteria, including many beneficial species such as *E. coli*, which are involved in a variety of metabolic processes in the body, including aiding in digestion and synthesis of essential nutrients. A decrease in anamorphic bacteria may affect the metabolic balance within the ear canal, reducing the production of protective substances by beneficial bacteria and further affecting the state of health of the ear canal (Rahimlou et al., 2021; Sun et al., 2021; Umeda et al., 2023). In conjunction with the previously discussed increase in the proportion of aerobic bacteria, we can speculate that under conditions of slow-acting otitis externa, changes in the microenvironment within the ear canal may have resulted in a decrease in *Actinomycetes* and *Ascomycetes*, while providing more space for anaerobic or other microorganisms tolerant of low-oxygen conditions to survive. This change in microbial composition not only affects the microbial diversity within the ear canal but may also influence the defense mechanisms and metabolic activity of the ear canal, thus playing a key role in the onset and persistence of slow-moving otitis externa. Therefore, the decrease in the relative abundance of *Actinobacteria* and *Proteobacteria* breaks the aerobic balance of the external auditory canal environment, leading to the outbreak of disease. In addition, *Actinobacteria* and *Proteobacteria* have carbohydrate metabolism capacity, which is consistent with the results predicted by PICRUSt2 function (Guo et al., 2023; Umeda et al., 2023).

A sample size of only 14 subjects per group can lead to certain limitations in the study’s findings. This is primarily reflected in the insufficient statistical power, which makes it difficult to detect effects unless they are exceptionally large. There may be a lack of sufficient data to identify true effects. Moreover, such a small sample size may result in findings that are not representative of a broader population, limiting the generalizability of the study. Estimates derived from this kind of sample may lack precision.

## 5 Conclusion

In this study, through analyzing the characteristics of ear canal flora in patients with chronic bacterial otitis externa, it was found that the diversity and abundance of ear canal flora in patients with chronic bacterial otitis externa were significantly lower than that in healthy controls, and the relative abundance of beneficial bacteria in ear canal was significantly lower than that in the control group. This provides a clue for clinical prevention of chronic bacterial otitis externa and also provides a direction for studying the prognosis of patients with chronic bacterial otitis externa. At the same time, the auricular flora spectrum provided by this study is of great significance for the comprehensive understanding of chronic bacterial otitis externa.

## Data Availability

The data presented in the study are deposited in the NCBI repository, accession number PRJNA1167757.
