# Supplementary material for: Changes functional prediction of ear canal flora in chronic bacterial otitis externa
Source: Front Cell Infect Microbiol. 2024 Oct 23;14:1434754. doi: 10.3389/fcimb.2024.1434754 (PMC11538054; doi:10.3389/fcimb.2024.1434754)
Supplement: Supplementary file 1 [file Table1.docx]

Supplementary Material

Changes and functional prediction of ear canal flora in chronic bacterial otitis externa

Tingting Duan^1†^, Zhiqun Li^1†^, Xiaoyong Han^2†^, Qichao Hong^3^, Yunan Yang^1^, Jinren Yan^1*^,Chengliang Xing^1*^

^1^ Department of Otolaryngology, Head and Neck Surgery, The First Affiliated Hospital, Hainan Medical University, Haikou 570102, China,

^2^Department of Otolaryngology, Linping District Hospital of Traditional Chinese Medicine, Hangzhou, Zhejiang 311100 , China,

^3^Department of Otorhinolaryngology Head and Neck Surgery, Hainan General Hospital (Hainan Affiliated Hospital of Hainan Medical University), Haikou 570311, China

Table S1.Summary of sequence number, base number and average sequence base number.

| Sample | Seq_num | Base_num | Mean_length |
| --- | --- | --- | --- |
| CB1 | 69925 | 28053447 | 401.193379 |
| CB2 | 41596 | 16079831 | 386.571569 |
| CB3 | 65076 | 26989961 | 414.745236 |
| CB4 | 56371 | 23582496 | 418.344468 |
| CB5 | 56830 | 24285390 | 427.333979 |
| CB6 | 138165 | 58776594 | 425.408707 |
| CB7 | 115865 | 47364598 | 408.791248 |
| CB8 | 66955 | 28516004 | 425.898051 |
| CB9 | 69268 | 29416546 | 424.677282 |
| CB10 | 57603 | 24700674 | 428.808812 |
| CB11 | 74508 | 31065041 | 416.935644 |
| CB12 | 91139 | 37492710 | 411.379431 |
| CB13 | 64531 | 27681891 | 428.970433 |
| CB14 | 63214 | 27102419 | 428.740769 |
| H1 | 79536 | 33326348 | 419.009606 |
| H2 | 62963 | 26336230 | 418.281054 |
| H3 | 280966 | 116304657 | 413.945662 |
| H4 | 58111 | 24120756 | 415.080725 |
| H5 | 190940 | 79825645 | 418.066644 |
| H6 | 79086 | 32598015 | 412.184394 |
| H7 | 62749 | 26656498 | 424.811519 |
| H8 | 64334 | 26981652 | 419.399571 |
| H9 | 58417 | 24981936 | 427.64839 |
| H10 | 74884 | 30898684 | 412.62064 |
| H11 | 61482 | 26356230 | 428.682053 |
| H12 | 62631 | 26862023 | 428.893407 |
| H13 | 62910 | 26953753 | 428.44942 |
| H14 | 64727 | 27738044 | 428.539002 |
